# A Bibliometric Review on *Candida auris* of the First Fifteen Years of Research (2009-2023)

**DOI:** 10.1155/2023/2385018

**Published:** 2023-10-12

**Authors:** Hiram Villanueva-Lozano, Alejandro I. Trejo-Castro, Diego Carrion-Alvarez, Sofía T. Lozano-Díaz, Antonio Martinez-Torteya

**Affiliations:** ^1^Internal Medicine Department, ISSSTE Regional Monterrey, Monterrey 64380, Nuevo Leon, Mexico; ^2^School of Medicine and Health Sciences, Tecnológico de Monterrey, Monterrey 64710, Nuevo Leon, Mexico; ^3^Bicultural Nursing Program, Instituto Humanístico de la Salud, Nuevo Laredo 88000, Tamaulipas, Mexico; ^4^Health Sciences, Universidad de Monterrey, San Pedro Garza García 66238, Nuevo Leon, Mexico; ^5^School of Engineering and Technology, Universidad de Monterrey, San Pedro Garza García 66238, Nuevo Leon, Mexico

## Abstract

**Introduction:**

*Candida auris* is a relatively novel pathogen first described in 2009 in Japan. It has increased its presence worldwide, becoming a public health concern due to its innate resistance to antifungals and outbreak potential.

**Methods:**

We performed a query using the word “*Candida auris”* from the Scopus database, further performing a bibliometric analysis with the open-source R package Bibliometrix.

**Results:**

907 original articles were retrieved, allowing us to map the principal authors, papers, journals, and countries involved in this yeast research, as well as analyze current and future trends and the number of published articles.

**Conclusion:**

*C. auris* will continue to be a pivotal point in fungal resistance research, either for a better understanding of its resistance and pathogenic mechanisms or for developing novel drugs.

## 1. Introduction

*Candida auris* was first described in 2009 when it was extracted from the external ear canal of a female inpatient in a hospital in Japan. Since then, there have been multiple reports around the globe of the emergence of this multi-drug-resistant yeast of the *Candida*/Clavispora clade closely related to *Candida haemulonii* and *Candida lusitaniae*. This fungal pathogen has expanded through six continents and multiple countries, causing nosocomial outbreaks with a fatality ratio as high as 72%. It represents the first example of an emerging pathogenic fungus caused by global warming [1–3].

There are many alarming features of the disease caused by this emerging fungal disease, such as its complex recognition by phenotypic and biochemical tests requiring more advanced techniques such as DNA sequencing and matrix-assisted laser desorption ionization-time of flight (MALDI-TOF) mass spectrometry to species-level identification; its thermotolerance, in which *C. auris* can grow at high temperatures > 40°C; its ability to tolerate high salt concentrations, both of which characteristics that contribute to its persistence and survival on abiotic surfaces for long periods; and its intrinsic resistance to one or more classes of antifungal drugs [2, 3].

Most of the *C. auris* isolates are resistant to fluconazole. However, five clades have been described as emerging simultaneously worldwide with subtle differences in their de facto antifungal resistance. *C*. *auris* South Asia clade I isolates are usually resistant to fluconazole, variably resistant to amphotericin B, and rarely to echinocandins. South American clade IV includes isolates with variable resistance to amphotericin B. South African clade III is commonly resistant to azoles. A fifth clade was reported in Iran as resistant to all three major classes of antifungals. Recently, independently from its intrinsic resistance, *C. auris* prolonged exposure to antifungals can lead to panfungal drug resistance. Transmission of pan-resistant strains has been documented in Texas and the District of Columbia (DC) in the United States of America (USA). This alarmingly growing resistance constitutes one of the main problems when dealing with this fungal disease [3–5].

In the context of the COVID-19 pandemic, multiple hospital outbreaks were described in specialized units. In addition, cases in Brazil, Colombia, Mexico, the USA, and many more countries were reported with a shift in prevalence of this disease from Asia to America and presenting with a mortality as high as 83% [2, 6–9].

Due to all this is important to keep documenting and studying formally the disease caused by this emergent fungal species, primarily because many of the reports of this disease are descriptive studies. Bibliometric analysis is a method for exploring and analyzing scientific data with the primary objective of presenting the scientific efforts made in studying a specific subject, often used to describe and predict tendencies for the upcoming years in specific research [10]. Here, we present to our knowledge the broader bibliometric analysis of information about *C. auris* presented in the literature.

## 2. Materials and Methods

### 2.1. Data Source and Search Strategy

Data were retrieved on 22 February 2023, from the Scopus database. The query used was “*Candida auris*” within the article title, its abstract, and its keywords for all the documents in the database (TITLE-ABS-KEY(“*Candida auris*”)). This means that we queried all publications where “*Candida auris”* as words must be together.

Documents were then filtered by document type, considering only original articles, and then by language, including only articles written in English (TITLE-ABS-KEY (“*Candida auris*”) AND (LIMIT-TO(DOCTYPE, “ar”)) AND (LIMIT-TO(LANGUAGE, “English”))). Quantitative and qualitative analyses, such as citation information (e.g., author(s), document title, and year), bibliographical information (e.g., affiliations and publisher), abstract and keywords (e.g., author keywords and index keywords), funding details, and other features, were extracted using the export document settings in the Scopus database.

### 2.2. Maps Based on Bibliographical Data

Most of the analyses carried out in this study were performed with the help of the open-source R package Bibliometrix [11].

## 3. Results

### 3.1. Overview

#### 3.1.1. Main Information

A total of 907 original articles on *C. auris* were found between 2009 and February 2023. Descriptive information regarding the collection can be consulted in Table 1. It is worth to mention that the annual growth rate is 22.93% and the document average age is of 2.76, giving us the understanding that the majority of the research in *C. auris* has been carried out in the last years and that there are strong collaborations between researchers, because only 37 articles, a 4.08% of the collection, are single-authored documents. Moreover, the average number of coauthors is 7.85, and nearly 1 in every 3 articles has an international coauthorship.

#### 3.1.2. Annual Scientific Production and Prediction

**Figure 1:**
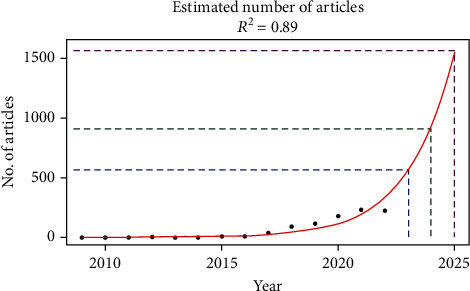
Estimation of original articles by year on *Candida auris*.

The year-wise distribution of these publications and the prediction of the number of articles for the following 3 years are shown in Figure 1. Between 2009 and 2014, there were only 7 original articles; then, the research interest increased over time. It can be expected that the research field would grow further soon; hence, to test this hypothesis, we fit an exponential regression model on the 2009-2022 data. The adjusted *R*^2^ was 0.89, this feature measures the relative predictive power of the exponential model, and the closer the value is to 1, the more accurate the model is. According to the regression analysis results, it was estimated that 552, 931, and 1572 original articles would be published in 2023, 2024, and 2025, respectively.

### 3.2. Sources

Table 2 shows the top 10 sources that generated most of the publications in the study, specifying the number of publications (NP), total number of citations (TC), h-index (h), the year of the first publication for that source (YFP), and the Journal Citation Reports 2021 impact factor and category.

We can see that these journals contribute 33% of the scientific knowledge on the matter. It is important to highlight that even though Emerging Infectious Diseases is the last in this table, it is the third one in total citations and the one with the highest impact factor. Further analysis on the primary focus of the most sources revealed that it is indeed an interdisciplinary research field. For instance, the source with the most publications, Antimicrobial Agents and Chemotherapy, deals with Pharmacology as well as Microbiology. Similarly, Journal of Fungi, Mycoses, and Medical Mycology cover Mycology, Dermatology, and Veterinary Sciences, respectively.

### 3.3. Authors

#### 3.3.1. Authors

The top 10 authors according to the NP are shown in Table 3. The features g and m stand for g-index and m-index, respectively. The g-index represents that given a set of articles ranked in decreasing order of the number of citations that they received; the g-index is the unique largest number such that the top g articles received together at least g^2^ citations. Meanwhile, the m-index is the h-index divided by the number of years that a scientist has been active and for that reason is more relevant to an earlier career researcher than the h-index. The affiliation information is generated by Scopus and is based on the most recent publication. It is worth highlighting the fact that half of these authors belong to the Centers for Disease Control and Prevention (CDC). This was to be expected because, according to our analyses, the affiliation with most publication is the CDC with 62, followed by the University of Debrecen (51), the University of Delhi (45), the University of the Witwatersrand (34), and the University of Wisconsin (29). Going back to the top 10 authors, 6 of them have affiliations to universities or research centers in the USA, while 2 of them have affiliations in India and 2 in Europe.

#### 3.3.2. Countries

The top 10 countries according to the NP are mentioned in Table 4. The worldwide scenario is led by the USA with 346 publications (38%). To analyze the international collaboration rate, publications were classified according to the country of affiliation of the corresponding author (CA). Records for the publications with a CA of each country were classified as either single country publication (SCP) or multiple country publication (MCP), and the MCP/CA ratio was calculated. Due to a report of missing data by Bibliometrix and inconsistencies in corresponding author information, we proceed to calculate these results without the software.

### 3.4. Documents

#### 3.4.1. Most Global Cited Documents

Out of the 907 original articles published in the 2009-2023 period on *C. auris*, the top ten articles that received the most citations are shown in Table 5. It is worth noting that even though Antimicrobial Agents and Chemotherapy had the highest number of citations per source, it does not appear in this table.

#### 3.4.2. Trending Topics

**Figure 2:**
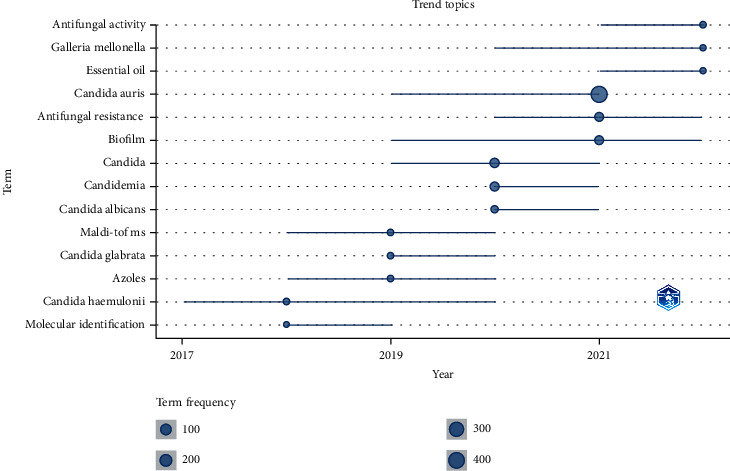
Trending topics.

In a bibliometric study, another important piece of information is to capture trending topics, so future research on *C. auris* could focus on emerging priorities for this disease. To uncover trending topics, the keywords provided by the authors in the most recent publications were analyzed. Results shown in Figure 2 indicate that the actual trending's are as follows: antifungal activity, *Galleria mellonella*, and essential oils. On the *x*-axis, we have the time span in years, and on the *y*-axis, we have the list of trending topics. The size of the circle indicates the frequency of the term, the position on the circle marks the year with most publications on that topic, and the line defines how long that term has been present.

### 3.5. Conceptual Structure

#### 3.5.1. Network Approach: Cooccurrence Network, Thematic Evolution, and Thematic Map

**Figure 3:**
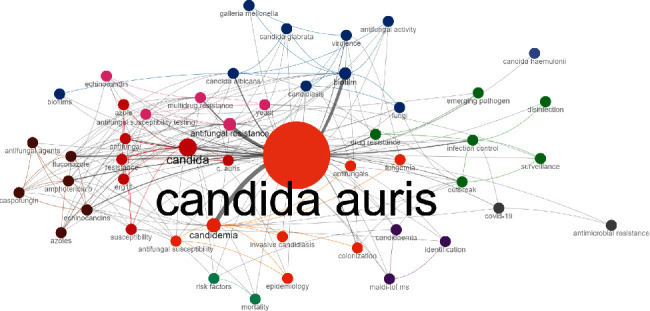
Author keyword cooccurrence network.

Figure 3 is a cooccurrence network, which is a collective interconnection of author keywords based on their presence in *C. auris* studies. A cooccurrence of two author keywords is when they appear in a particular article which forms an edge. The thicker the edge means a higher frequency of words cooccurring. The different colors used in the plot indicate word clusters. The clusters and their nodal positions were based on the values of the features betweenness, closeness, and page rank using a walk trap clustering method.

**Figure 4:**
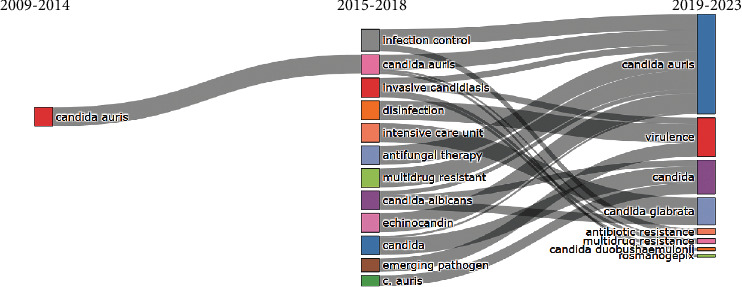
Thematic evolution map.

Figure 4 is a thematic evolution map that indicates the evolution of author keywords in three different stages (2009-2014, 2015-2018, and 2019-2023). As we mentioned before from 2009 to 2014, there were only 7 original articles, and obviously, the keyword that most appeared was “*candida auris*”. At the time, when the research on the subject was evolving and being more numerous, it began to diversify, generating more keywords. An important keyword is “multidrug resistance” as this shows up in the last two stages. At the last stage, “fosmanogepix” appears which is an experimental antifungal drug being developed by Pfizer.

**Figure 5:**
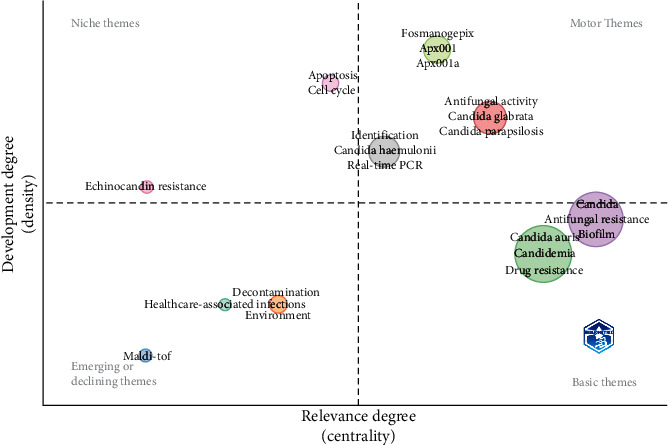
Thematic map.

Figure 5 shows a thematic map which classifies author keyword groupings according to the quadrant in which they are found. The *x*-axis corresponds to the relevance and the *y*-axis to the development of the topic. At the upper right quadrant are the motor themes, at the lower right quadrant are the fundamental themes, on the left in the lower corner are the emerging or disappearing themes, and those in the upper left are the extremely specialized topics.

#### 3.5.2. Factorial Analysis

**Figure 6:**
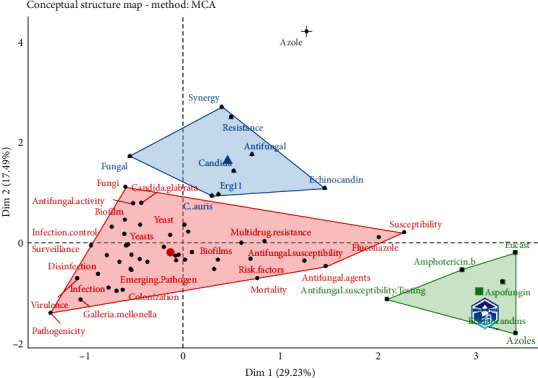
Conceptual structure map.

**Figure 7:**
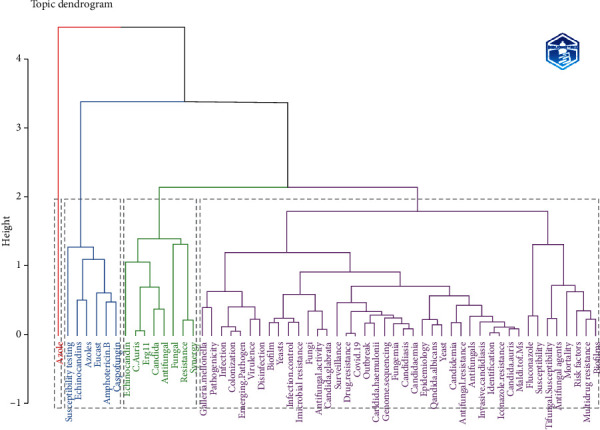
Dendrogram conceptual structure.

Figure 6 is a conceptual structure map that represents relations among keywords in a set of publications. Through multiple correspondence analysis (MCA), a conceptual structure of the field can be created and *k*-means clustering allows to find clusters of documents that discuss similar concepts.  Figure 7 is the dendrogram of the author keywords used in Figure 6; this diagram shows the hierarchical relationships between words.

#### 3.5.3. Intellectual Structure

**Figure 8:**
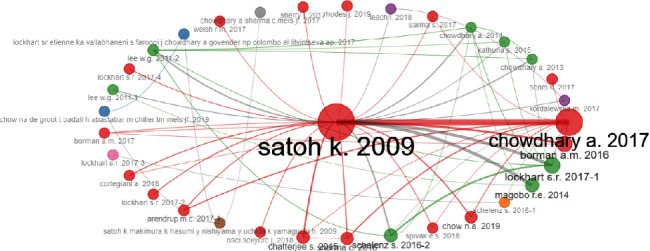
Cocitation network.

Figure 8 is a cocitation network and represents the frequency with which two documents are cited together by other documents. If at least one other document cites two documents, these documents are said to be cocited. The minimum number of edges is 15 to appear in this network. As we can appreciate in the plot, Satoh [1], the first article ever on *C. auris*, is at the center of the plot. This paper has a strong edge with the Chowdhary [12] review article about the rapid fungal infections globally, which means that they are frequently cocited. The colors represent clusters.

#### 3.5.4. Social Structure

**Figure 9:**
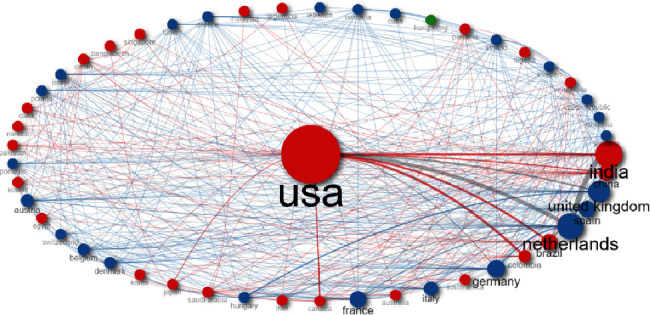
Country collaboration network.

**Figure 10:**
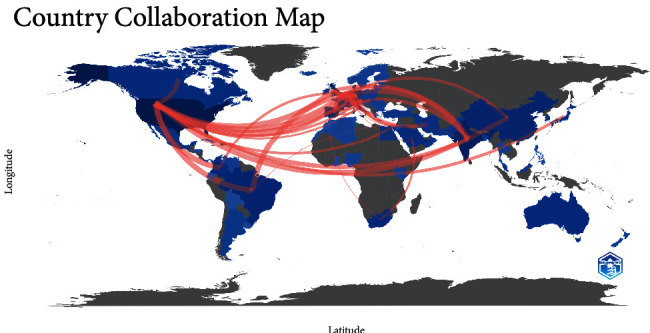
Country collaboration world map.

Figure 9 shows the country collaboration network. Here, we can see 2 main clusters: the blue cluster indicates relationships mainly between European countries while the red cluster highlights the relationships between USA and Asian, Arabic, and Latin American countries.  Figure 10 is similar as it indicates country collaboration where the pink color line represents the connection between countries and its thickness identifies the degree of collaboration (minimum edge is 5). The thicker the line, the stronger the collaboration rate. The major collaborations were India-Netherlands 26, USA-Netherlands 22, Brazil-Netherlands 17, USA-Colombia 17, and USA-Brazil 16.

## 4. Discussion

*C. auris* infection has increased over the years, and its poor prognosis, high mortality rate, and rapid spread have drawn the attention of researchers, making it a trending topic. This bibliometric study retrieved 907 original articles published between 2009 and 2023. According to the exponential fitting curve, the annual number of publications showed an upward trend, rocketing up in the first half of the period, especially after 2015. According to the expected function model, the number of publications in 2023 will increase to 552 (or ~552), while in 2025, several 1572 are expected (or ~1572) which will be a higher number of publications in this field.

A total of 285 journals reported research in *C. auris*, and the top 10 are presented in Table 2. Antimicrobial Agents and Chemotherapy rank first with 64 publications (7.06%), followed by Journal of Fungi (58, 6.39%) and Mycoses (3.42%). Among the top 10 academic journals, according to Scimago Journal & Country Rank, five are from the USA, three are from the United Kingdom, and two are from Switzerland, showing a solid research foundation in these countries. Eight journals have an impact factor > 5, including Emerging Infectious Diseases (16.126), Journal of Clinical Microbiology (11.677), mBio (7.786), Infection Control and Hospital Epidemiology (6.520), Frontiers in Microbiology (6.064), Antimicrobial Agents and Chemotherapy (5.938), Journal of Fungi (5.724), and mSphere (5.029). Web of Science Journal Citation Ranking is a tool that measures journals' scientific impact, influence, and prestige based on the average number of weighted citations [13]. Nine journals (Antimicrobial Agents and Chemotherapy, Journal of Fungi, Mycoses, Journal of Clinical Microbiology, mBio, Medical Mycology, Frontiers in Microbiology, Infection Control and Hospital Epidemiology, and Emerging Infectious Diseases) are considered of Q1 quality with far-reaching development prospects.

Table 3 shows the top 10 authors in terms of publication volume and centrality. The most prolific author, with 52 papers in this field, is Jacques F. Meis from the Canisius Wilhelmina Hospital at the Netherlands, with a h-index of 92, who has published over 599 articles according to Scopus, in which his most contributed topics between 2017 and 2021 were as follows: isavuconazonium, antifungal agents, and voriconazole (25 documents); *Candida* spp., antifungal agents, and voriconazole (12 documents); and *Cryptococcus gattii*, flucytosine, and *Cryptococcus neoformans* (6 documents). The second-ranked author was Abhay Sadashiv Chowdhary, a professor in Vallabhbhai Patel Chest Institute in New Delhi, India, with a h-index of 17, who has published over 114 articles, in which his most contributed topics between 2017 and 2021 were as follows: human influenza, oseltamivir, and laninamivir (3 documents); dengue, antibody-dependent enhancement, and flavivirus (2 documents); and mutation, isoniazid, and ethambutol (2 documents); this author, even having fewer articles compared to the first author, has received considerably higher citations compared to all authors in the top 10. The third-ranked author was Shawn R. Lockhart from the CDC, with a h-index of 67, who has published over 243 articles, in which his most contributed topics between 2017 and 2021 were as follows: *Candida spp.*, antifungal agents, and voriconazole (33 documents); isavuconazonium, antifungal agents, and voriconazole (10 documents); and candidemia, invasive candidiasis, and antifungal agents (9 documents). It is worth mentioning that within this top 10 authors, 5 of them are affiliated with the CDC; this is to be expected since, being an emerging infection, a national agency like this one, in charge of protecting health and safety, injuries, and disabilities in the USA and around the world, will see further development in this area.

These publications came from 77 countries. The top ten countries are distributed in Europe, America, and Asia, mainly in Europe (*n* = 6) (Table 4). Among them, the country with the largest number of publications is the USA (*n* = 346), followed by India (*n* = 129), United Kingdom (*n* = 82), and Netherlands (*n* = 75). The top 10 authors are located in 4 countries, with three-fifths of them located in the USA. In the bibliometric study by Ramírez-Malule et al. (2020) which covers research on *C. auris* between 2009 and 2018, China was in the tenth place among the countries with the most studies related to C. *auris*. Now, China has caught up to where the UK once stood, having advanced exponentially since 2018 from a total of 7 publications to 60; it is important to remember that in contrast to the previous paper, we only considered original articles [14].

Now, it is important to look at the collaborations between the different countries. With Bibliometrix software, it was found that the main collaborations were between the following countries: India-Netherlands (*n* = 26), USA-Netherlands (*n* = 22), Brazil-Netherlands (*n* = 17), USA-Colombia (*n* = 17), and USA-Brazil (*n* = 16); these countries were also the most productive in terms of publications relating to *C. auris*.

In the field of research, there are different factors for the existence of advances in medical research; one of them is academic capability, which is determined by the economic status of a country and governmental expenditure on healthcare [15]. One country that invests the most in the research area is the USA, with USD10,784USD10,202 per resident [16], which could explain in a certain way why it is the country with the most publications on *C. auris*.

Further, in the analysis, the top cited publications have Lockhart et al.; this paper analyzed 54 isolates from 5 countries, not only collecting clinical data of the patients but also performing molecular techniques into the strains to know antimycotic resistance and whole genome sequencing, demonstrating that each geographical region has unique clades associated with azole resistance [17]. The second most cited paper corresponds to Satoh et al., which describes the first case reported in Japan in 2009 [1]. Schelenz et al., in 2017, published the first hospital outbreak of C. *auris* [18]. The other seven that conform to the top ten most cited can be divided into outbreak reports [19, 20], reviews [21, 22], molecular methods to asses resistance and identification [23, 24], and a global surveillance report [25]. Interestingly at the moment of publication of this latest article, only 6 cases of C. *auris* have been reported. Therefore, it is slightly mentioned in the document. Furthermore, the three top cited papers have kept this position since a previous bibliometric was published in 2020 by Ramírez-Malule et al. [14].

Regarding the trending topics, it allows us to picture the changes through time in strain research. C. *auris* is a young pathogen allowing us to map its research efforts and critical themes. Considering previous work, we limited our trending topics to the last five years. Upon the start of 2017-2018, we saw a decline in molecular identification since it became a standard part of the research and a slight spike regarding *C. haemulonii*; this species represents another emerging pathogen with significant antimycotic resistance worldwide [26]. Other pathogens with the same concerns can be found as trending topics in the upcoming years, such as *Candida glabrata* from 2019 to 2020. Another topic that peaked during 2019 was the “Azoles”; referring to the papers examining resistance, another topic of interest was “MALDI-TOF,” which refers to matrix-assisted laser desorption/ionization-time of flight. While this technique was developed in 1987, it quickly became a potential tool for microbial identification [27]. In 2015, this method started identifying C. *auris* in research and clinical environments when cultures were proved insufficient [28]. Antifungal resistance still is a theme of concern. Finally, in 2022, the trending topics include essential oils; this research branch is currently exploring aromatic plants such as Thymus or Cinnamomum [29, 30]. The oils have shown antifungal activity in resistant strains [31]. Another trending topic is “*galleria mellonella*”; these larvae are currently used as an in vivo model for examining C. *auris* virulence [32]. Finally, “antifungal activity” is another set of words currently trending, mainly due to the novel antifungals researched for this fungus and other mycoses.

The conceptual structure allows us to make visible research revolving around this pathogen. Divided into colored clusters, we can see how blue, green, pink, red, and brown dots revolt around the resistance and virulence of the strain. In contrast, orange ones focus on outbreaks and places where they occur, such as intense care units (ICUs). Other dots appear less related, like the light blue with mortality and gray ones, which link this strain with the COVID-19 pandemic and antimicrobial resistance. Similarly, the thematic map allows us to see how multiple branches came from a single term, “C. *auris*,” in 2009. From 2015 to 2018, they mainly related once more to outbreaks, how to prevent them, and resistance. Then, it changed again from 2019 to 2023 when the main topics now revolve around virulence and other concerning pathogens. Novel drugs are being researched for their treatment.

All of this past and future can be better understood with the thematic map, showcasing the pathogen's story. We find declining themes such as MALDI-TOF and decontamination or environment in the lower left quadrant. These themes were relevant in the past but are now common in publications. Niche themes can be found in the upper left quartile. The topics here include echinocandin resistance, which is a motive of concern since some strains have shown innate resistance to all echinocandins.

Furthermore, the test with caspofungin sometimes exhibits an eagle effect which can be confusing in determining resistance [33]. Another niche theme is the cell cycle to understand the yeast's physiology better. Moving into the lower right quartile, we find basic themes in *C. auris* research, such as the name of the pathogen and keywords such as biofilm, resistance, and candidemia. Finally, the upper right quartile showcases the motor themes and topics currently being pushed and will outline future research in this theme. Here, we find a cluster of keywords related to real-time PCR and *C. haemulonii*. The other method is to identify *C. auris* and allow for a better delineation from *C. haemulonii*, a close species complex. Furthermore, commercially available kits allow the identification in clinical settings that lack technology, like the MALDI-TOF [34].

Other keywords encompass other candida species, such as *C. glabrata* and *C. parapsilosis*, which are monitored for multidrug resistance [35, 36]. Finally, the last cluster focuses on the novel antifungal that is being researched. The new drug “fosmanogepix,” also known as APX001, has a novel mechanism of action targeting the glycosylphosphatidylinositol-anchored protein by inhibiting a fungal enzyme preventing the adherence of the fungal to surfaces, it is also the first broad-spectrum antifungal, and it currently has the fast track status by the US Food and Drug Administration (FDA) for various invasive fungal diseases [37]. While not mentioned in our analysis, it is worth mentioning that other drugs, such as ibrexafungerp, olorofim, opelconazole, and rezafungin, are being developed for multi-drug-resistant fungi.

The conceptual structure also complements this perspective. It was divided into four clusters; the red one revolves around virulence factors and properties of the pathogen to avoid immune responses such as biofilms. It also includes risk factors, colonization, and multidrug resistance. Conversely, the green cluster focuses on antifungal drugs like caspofungin and amphotericin B and drug groups like azoles and echinocandins. The blue cluster includes a small number of words, such as *candida*, echinocandin, and fungal. One of the most interesting findings is “erg11,” a gene described in *Candida* species and codes for azole resistance [38].

Finally, the cocitation network, while challenging to analyze each article by itself, however, is remarkable that the paper published by Satoh in 2009 stays at the center as being the most cocited in all *C. auris* literature; [1] it has strong relationships with other published papers including a review of the pathogen published by Chowdhary et al. in 2017 [12] and an original article published by Borman et al. in 2016 which compared the pathogenicity of *C. auris* isolates, as well as other key candida species [39]. Finally, another article with a strong relationship was published in 2014 by Magobo et al.; this is a letter to the editor describing four isolates from South Africa which were molecularly identified and compared to other strains [40]. Overall, the cocitation network allows us to see that current research is growing and allowing other papers to be created.

Future tendencies in C. auris revolve around the search for new antifungal drugs [41, 42] that allow clinicians to increase the effectivity of treatments and reduce adverse effects in patients, while it is still in early developing, new antifungal families such as triterpenoids [43] and olorofim [44] strengthen existing antifungals such as azoles and echinocandins, and the research of new delivery methods like nanoparticles will be on the center of fungal research [45, 46].

However, research will also focus on fully understanding how it works, molecular studies into resistant genes, and how they continue to evolve, and the use of molecular sciences to fully understand its pathogenetic abilities will open doors in the mycology field. *In vitro* studies using various agar combinations have focused on understanding the role and characteristics of biofilm formation, as well as the ability of the yeast to survive high temperatures and enzyme secretion as pathogenic mechanisms [47]. On the other hand, *in vivo* models have also increased; with *Galleria mellonella* as the some term related this area that appeared in our results, however, the use of murine models with knockout genes also allow us to examine how different strains work within the immune system of a live being [48].

Finally, and due to current conditions, clinical research and epidemiology also have a road ahead; they must both understand factors such as the on-site setting of transmission and the actions that can be taken to control outbreaks and reduce infection rates, as well as understand the complications, coinfections and risk factors of patients by understanding from an on-site setting the transmission and the actions that can be taken to control the outbreaks and diminish infection rates; as well as understand the complications, coinfections and risk factors of the patients. The implications of the bibliometric data limit this article. We can only predict, to a certain extent, future tendencies.

## 5. Conclusions

This pathogen, *C. auris*, will continue to be a concern in clinical settings and research. Its capability to resist multiple antifungals prompts scientists and the pharmaceutical industry to develop novel treatments and more accurate and accessible identification methods. We also expect an increase in clinical trials for new antifungals. This work presents a trending analysis of topics related to *C. auris*. While some may not be pursued in the future, others will be the motor themes for researchers worldwide. What is certain is that this strain represents a challenge for hospitals and doctors alike since it continues to increase worldwide.

## Figures and Tables

**Table 1:** Summary of descriptive information on the original articles collection found from 2009 to 2023.

Feature	Explanation	Count
*Main information about data*		
Documents	Total number of scientific publications	907
Sources	The frequency distribution of sources as journals	285
Annual growth rate (%)	The average increase in the number of documents over a year	22.93
Document average age	Average age of a document given in years	2.76
Average citations per doc	The average number of quotes in each article	23.79
References	Total number of references	32163
*Document contents*		
Keywords plus (ID)	Total number of word or phrases that frequently appear in the title of an article's references	6298
Author's keywords (DE)	Total number of keywords	1849
*Authors*		
Authors	Total number of authors	4624
Authors of single-authored docs	The number of single authors per articles	35
*Authors collaboration*		
Single-authored docs	Total number of single-authored documents	37
Coauthors per doc	The average number of coauthors in each document	7.85
International coauthorships (%)	The average number of articles with international collaboration	32.19

**Table 2:** List of journals with the highest number of publications on the subject in our collection.

Source	NP (%)	TC	h	YFP	JCR 2021	
Antimicrobial Agents and Chemotherapy	64 (7.06)	1830	22	2017	5.938	Q1 Pharmacology Q2 Microbiology

Journal of Fungi	58 (6.39)	551	12	2018	5.724	Q1 Mycology Q2 Microbiology

Mycoses	31 (3.42)	901	14	2016	4.931	Q1 Dermatology Q2 Mycology

Journal of Clinical Microbiology	28 (3.09)	1693	16	2011	11.677	Q1 Microbiology

mBio	23 (2.54)	682	13	2018	7.786	Q1 Microbiology

Medical Mycology	23 (2.54)	547	10	2010	3.747	Q1 Veterinary Sciences Q2 Mycology Q3 Infectious Diseases

mSphere	23 (2.54)	706	12	2016	5.029	Q2 Microbiology

Frontiers in Microbiology	22 (2.43)	223	9	2016	6.064	Q1 Microbiology

Infection Control and Hospital Epidemiology	18 (1.98)	395	9	2017	6.520	Q1 Public, Environmental, & Occupational Health Q2 Infectious Diseases

Emerging Infectious Diseases	16 (1.76)	1339	12	2013	16.126	Q1 Immunology Q1 Infectious Diseases

**Table 3:** List of authors with the highest number of publications on the subject in our collection.

Author	Affiliation	NP	h	g	m	TC	YFP
Meis, J. F.	Canisius Wilhelmina Ziekenhuis, Nijmegen, Netherlands	52	23	52	2.091	3961	2013
Chowdhary, A.	Vallabhbhai Patel Chest Institute, New Delhi, India	43	27	43	2.455	4013	2013
Lockhart, S. R.	Centers for Disease Control and Prevention, Atlanta, United States	31	20	31	2.500	2509	2016
Berkow, E. L.	Centers for Disease Control and Prevention, Atlanta, United States	24	16	24	2.000	2430	2016
Litvintseva, A. P.	Centers for Disease Control and Prevention, Atlanta, United States	21	14	21	1.750	2054	2016
Chakrabarti, A.	Postgraduate Institute of Medical Education & Research, Chandigarh, India	21	11	21	1.222	863	2015
Borman, A. M.	UK Health Security Agency, London, United Kingdom	19	11	19	1.375	844	2016
Vallabhaneni, S.	Centers for Disease Control and Prevention, Atlanta, United States	18	13	18	1.625	1723	2016
Forsberg, K.	Centers for Disease Control and Prevention, Atlanta, United States	18	11	18	1.833	791	2018
Chaturvedi, S.	Wadsworth Center for Laboratories and Research, Albany, United States	18	10	18	1.250	704	2016

**Table 4:** List of countries with the highest number of publications on the subject in our collection.

Country	NP	CA	SCP	MCP	MCP/CA ratio
United States of America	346	256	190	66	0.258
India	129	87	61	26	0.299
United Kingdom	82	42	30	12	0.286
Netherlands	75	19	2	17	0.895
China	60	46	31	15	0.326
Brazil	56	30	15	15	0.500
Spain	54	43	34	9	0.209
Germany	44	30	13	17	0.567
France	37	15	7	8	0.533
Italy	35	25	19	6	0.240

**Table 5:** Publications with the highest number of citations.

Title	First author	Journal	YP	TC	TC per year	Normalized TC
Simultaneous emergence of multidrug-resistant *Candida auris* on 3 continents confirmed by whole-genome sequencing and epidemiological analyses	Lockhart, S. R.	Clinical Infectious Diseases	2017	780	111.43	6.31
*Candida auris* sp. nov., a novel ascomycetous yeast isolated from the external ear canal of an inpatient in a Japanese hospital	Satoh, K.	Microbiology & Immunology	2009	597	39.80	1.00
First hospital outbreak of the globally emerging *Candida auris* in a European hospital	Schelenz, S.	Antimicrobial Resistance & Infection Control	2016	430	53.75	2.63
Multidrug-resistant *Candida auris* misidentified as *Candida haemulonii*: characterization by matrix-assisted laser desorption ionization–time of flight mass spectrometry and DNA sequencing and its antifungal susceptibility profile variability by Vitek 2, CLSI broth microdilution, and Etest method	Kathuria, S.	Journal of Clinical Microbiology	2015	331	36.78	2.38
Invasive candidiasis	Pappas, P. G.	Nature Reviews Disease Primers	2018	330	55.00	5.94
Multidrug-resistant *Candida*: epidemiology, molecular mechanisms, and treatment	Arendrup, M. C.	The Journal of Infectious Diseases	2017	309	44.14	2.50
First three reported cases of nosocomial fungemia caused by *Candida auris*	Lee, W. G.	Journal of Clinical Microbiology	2011	285	21.92	1.00
Incidence, characteristics and outcome of ICU-acquired candidemia in India	Chakrabarti, A.	Intensive Care Medicine	2015	279	31.00	2.00
Twenty years of the SENTRY antifungal surveillance program: results for *Candida* species from 1997–2016	Pfaller, M. A.	Open Forum Infectious Diseases	2019	274	54.80	9.22
A multicentre study of antifungal susceptibility patterns among 350 *Candida auris* isolates (2009–17) in India: role of the *ERG11* and *FKS1* genes in azole and echinocandin resistance	Chowdhary, A.	Journal of Antimicrobial Chemotherapy	2018	271	45.17	4.88

## Data Availability

Data used in the preparation of this article were obtained from the Scopus database. Further inquiries can be directed to the corresponding author.

## References

[1] Satoh K., Makimura K., Hasumi Y., Nishiyama Y., Uchida K., Yamaguchi H. (2009). *Candida auris* sp. nov., a novel ascomycetous yeast isolated from the external ear canal of an inpatient in a Japanese hospital. *Microbiology and Immunology*.

[2] Thakur S. (2022). State of the globe: *Candida auris*-a global healthcare threat. *Journal of Global Infectious Diseases*.

[3] Du H., Bing J., Hu T., Ennis C. L., Nobile C. J., Huang G. (2020). *Candida auris*: epidemiology, biology, antifungal resistance, and virulence. *PLOS Pathogens*.

[4] Jacobs S. E., Jacobs J. L., Dennis E. K. (2022). *Candida auris* pan-drug-resistant to four classes of antifungal agents. *Antimicrobial Agents and Chemotherapy*.

[5] Dang T., Bassett J., Souri A. (2022). Transmission of pan-resistant and echinocandin-resistant Candida auris between healthcare facilities during the COVID-19 pandemic. *American Journal of Infection Control*.

[6] Vaseghi N., Sharifisooraki J., Khodadadi H. (2022). Global prevalence and subgroup analyses of coronavirus disease (COVID-19) associated *Candida auris* infections (CACa): a systematic review and meta-analysis. *Mycoses*.

[7] de Almeida J. N., Francisco E. C., Hagen F. (2021). Emergence of Candida auris in Brazil in a COVID-19 intensive care unit. *Journal of Fungi*.

[8] Prestel C., Anderson E., Forsberg K. (2021). *Candida auris* outbreak in a COVID-19 specialty care unit — Florida, July–August 2020. *MMWR. Morbidity and Mortality Weekly Report*.

[9] Villanueva-Lozano H., Treviño-Rangel R. . J., González G. M. (2021). Outbreak of Candida auris infection in a COVID-19 hospital in Mexico. *Clinical Microbiology and Infection*.

[10] Trejo-Castro A. I., Carrion-Alvarez D., Martinez-Torteya A., Rangel-Escareño C. (2022). A bibliometric review on gut microbiome and Alzheimer’s disease between 2012 and 2021. *Frontiers in Aging Neuroscience*.

[11] Aria M., Cuccurullo C. (2017). Bibliometrix: an R-tool for comprehensive science mapping analysis. *Journal of Informetrics*.

[12] Chowdhary A., Sharma C., Meis J. F. (2017). *Candida auris*: a rapidly emerging cause of hospital-acquired multidrug-resistant fungal infections globally. *PLoS Pathogens*.

[13] Falagas M. E., Kouranos V. D., Arencibia-Jorge R., Karageorgopoulos D. E. (2008). Comparison of SCImago journal rank indicator with journal impact factor. *The FASEB Journal*.

[14] Ramírez-Malule H., Gómez-Ríos D., López-Agudelo V. A. (2020). *Candida auris*: a bibliometric analysis of the first ten years of research (2009–2018). *Journal of Applied Pharmaceutical Science*.

[15] Raghupathi V., Raghupathi W. (2020). Healthcare expenditure and economic performance: insights from the United States data. *Frontiers in Public Health*.

[16] Statista Research Department (2023). *Health expenditures in the U.S. – statistics & facts*.

[17] Lockhart S. R., Etienne K. A., Vallabhaneni S. (2017). Simultaneous emergence of multidrug-resistant *Candida auris* on 3 continents confirmed by whole-genome sequencing and epidemiological analyses. *Clinical Infectious Diseases*.

[18] Schelenz S., Hagen F., Rhodes J. L. (2016). First hospital outbreak of the globally emerging *Candida auris* in a European hospital. *Antimicrobial Resistance and Infection Control*.

[19] Chakrabarti A., Sood P., Rudramurthy S. M. (2015). Incidence, characteristics and outcome of ICU-acquired candidemia in India. *Intensive Care Medicine*.

[20] Lee W. G., Shin J. H., Uh Y. (2011). First three reported cases of nosocomial fungemia caused by *Candida auris*. *Journal of Clinical Microbiology*.

[21] Pappas P. G., Lionakis M. S., Arendrup M. C., Ostrosky-Zeichner L., Kullberg B. J. (2018). Invasive candidiasis. *Nature Reviews Disease Primers*.

[22] Arendrup M. C., Patterson T. F. (2017). Multidrug-resistant Candida: epidemiology, molecular mechanisms, and treatment. *The Journal of Infectious Diseases*.

[23] Chowdhary A., Prakash A., Sharma C. (2018). A multicentre study of antifungal susceptibility patterns among 350 Candida auris isolates (2009–17) in India: role of the ERG11 and FKS1 genes in azole and echinocandin resistance. *Journal of Antimicrobial Chemotherapy*.

[24] Kathuria S., Singh P. K., Sharma C. (2015). Multidrug-resistant *Candida auris* misidentified as *Candida haemulonii*: characterization by matrix-assisted laser desorption ionization–time of flight mass spectrometry and DNA sequencing and its antifungal susceptibility profile variability by Vitek 2, CLSI broth microdilution, and Etest method. *Journal of Clinical Microbiology*.

[25] Pfaller M. A., Diekema D. J., Turnidge J. D., Castanheira M., Jones R. N. (2019). Twenty years of the SENTRY antifungal surveillance program: results for Candida species from 1997–2016. *Open Forum Infectious Diseases*.

[26] Colombo A. L., Júnior J. N. A., Guinea J. (2017). Emerging multidrug-resistant Candida species. *Current Opinion in Infectious Diseases*.

[27] Singhal N., Kumar M., Kanaujia P. K., Virdi J. S. (2015). MALDI-TOF mass spectrometry: an emerging technology for microbial identification and diagnosis. *Frontiers in Microbiology*.

[28] Abdolrasouli A., Fraser M. A. (2022). *Candida auris identification and profiling by MALDI–ToF mass spectrometry*.

[29] Ribeiro R., Fernandes L., Costa R. (2022). Comparing the effect of *Thymus* spp. essential oils on *Candida auris*. *Industrial Crops and Products*.

[30] Tran H. N. H., Graham L., Adukwu E. C. (2020). In vitro antifungal activity of *Cinnamomum zeylanicum* bark and leaf essential oils against *Candida albicans* and *Candida auris*. *Applied Microbiology and Biotechnology*.

[31] Fernandes L., Ribeiro R., Costa R., Henriques M., Rodrigues M. E. (2022). Essential oils as a good weapon against drug-resistant *Candida auris*. *Antibiotics*.

[32] Romera D., Aguilera-Correa J.-J., García-Coca M. (2020). The *Galleria mellonella* infection model as a system to investigate the virulence of *Candida auris* strains. *Pathogens and Disease*.

[33] Kordalewska M., Lee A., Park S. (2018). Understanding echinocandin resistance in the emerging pathogen *Candida auris*. *Antimicrobial Agents and Chemotherapy*.

[34] Lockhart S. R., Lyman M. M., Sexton D. J. (2022). Tools for detecting a “superbug”: updates on *Candida auris* testing. *Journal of Clinical Microbiology*.

[35] Pristov K. E., Ghannoum M. A. (2019). Resistance of Candida to azoles and echinocandins worldwide. *Clinical Microbiology and Infection*.

[36] Healey K. R., Perlin D. S. (2018). Fungal resistance to echinocandins and the MDR phenomenon in *Candida glabrata*. *Journal of Fungi*.

[37] Hoenigl M., Sprute R., Egger M. (2021). The antifungal pipeline: fosmanogepix, ibrexafungerp, olorofim, opelconazole, and rezafungin. *Drugs*.

[38] Xiang M.-J., Liu J.-Y., Ni P.-H. (2013). *Erg11* mutations associated with azole resistance in clinical isolates of *Candida albicans*. *FEMS Yeast Research*.

[39] Borman A. M., Szekely A., Johnson E. M. (2016). Comparative pathogenicity of United Kingdom isolates of the emerging pathogen *Candida auris* and other key pathogenic *Candida* species. *MSphere*.

[40] Magobo R. E., Corcoran C., Seetharam S., Govender N. P. (2014). *Candida auris* –associated candidemia, South Africa. *Emerging Infectious Diseases*.

[41] Singh A., Singh K., Sharma A. (2023). 1,2,3-Triazole derivatives as an emerging scaffold for antifungal drug development against *Candida albicans*: a comprehensive review. *Chemistry & Biodiversity*.

[42] Kaur J., Nobile C. J. (2023). Antifungal drug-resistance mechanisms in *Candida* biofilms. *Current Opinion in Microbiology*.

[43] Bhattacharya S. P., Karmakar S., Acharya K., Bhattacharya A. (2023). Quorum sensing inhibition and antibiofilm action of triterpenoids: an updated insight. *Fitoterapia*.

[44] Wiederhold N. P. (2020). Review of the novel investigational antifungal olorofim. *Journal of Fungi*.

[45] Huang T., Li X., Maier M., O’Brien-Simpson N. M., Heath D. E., O’Connor A. J. (2023). Using inorganic nanoparticles to fight fungal infections in the antimicrobial resistant era. *Acta Biomaterialia*.

[46] Li Y., Zhang P., Li M. (2023). Application and mechanisms of metal-based nanoparticles in the control of bacterial and fungal crop diseases. *Pest Management Science*.

[47] Ahmad S., Alfouzan W. (2021). *Candida auris*: epidemiology, diagnosis, pathogenesis, antifungal susceptibility, and infection control measures to combat the spread of infections in healthcare facilities. *Microorganisms*.

[48] Forgács L., Borman A. M., Prépost E. (2020). Comparison of *in vivo* pathogenicity of four *Candida auris* clades in a neutropenic bloodstream infection murine model. *Emerging Microbes & Infections*.

